# Supplementary material for: Revisiting p53 Immunohistochemical Staining and Its Prognostic Implications in Advanced EGFR-Mutated Lung Adenocarcinoma
Source: Cancers (Basel). 2025 Nov 5;17(21):3577. doi: 10.3390/cancers17213577 (PMC12608574; doi:10.3390/cancers17213577)
Supplement: Supplementary file 1 [file cancers-17-03577-s001.zip › cancers-3943875-supplementary.pdf]

Figure S1.

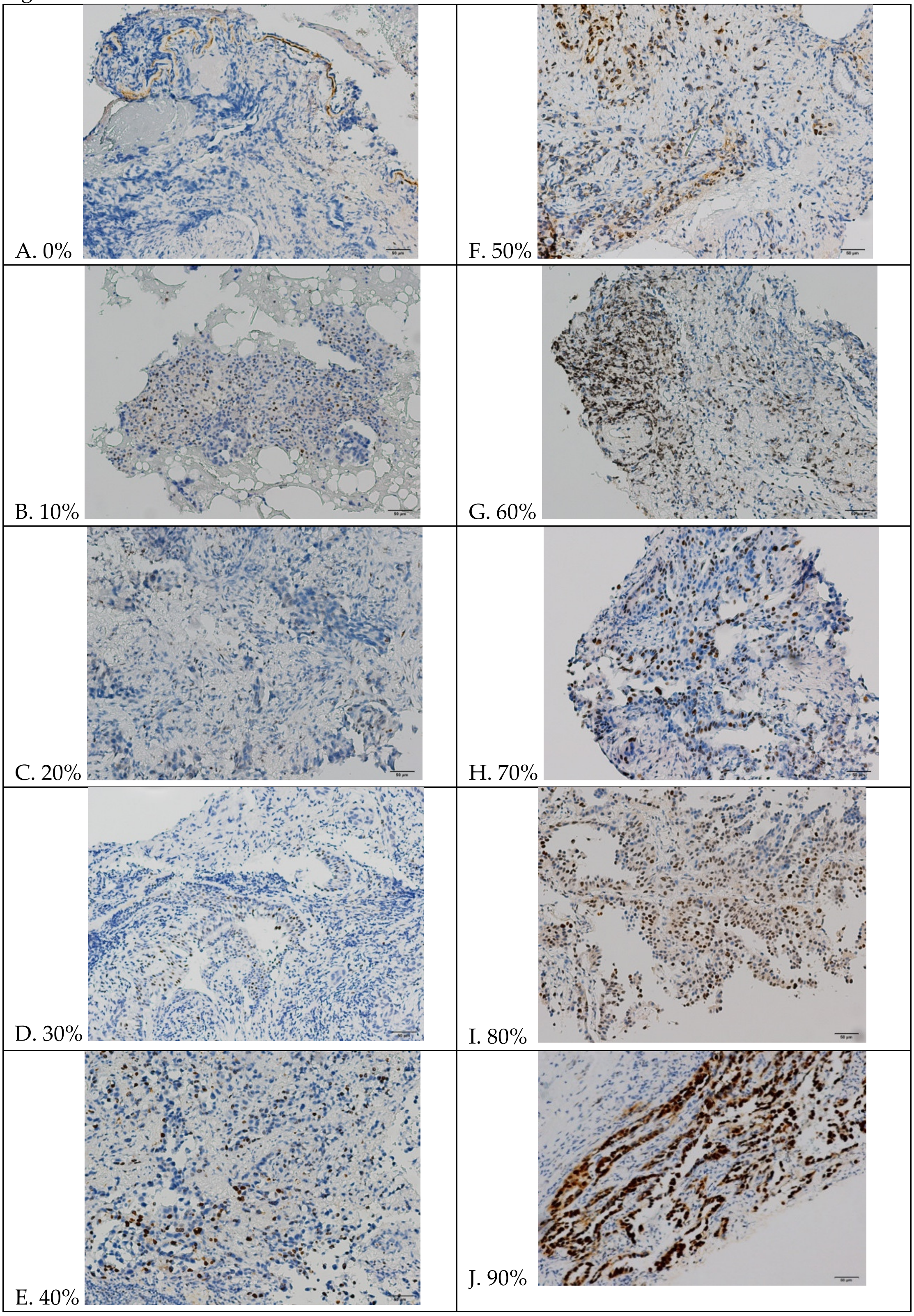

Nuclear p53 immunostaining was defined as the proportion of positive tumor cells (%). IHC staining of nuclear p53 with a p53 monoclonal antibody (Clone: DO-7, Code M7001, dilution 1/50, Dako); scale 50 μm. A to J represent 0% to 90% staining.

Figure S2.

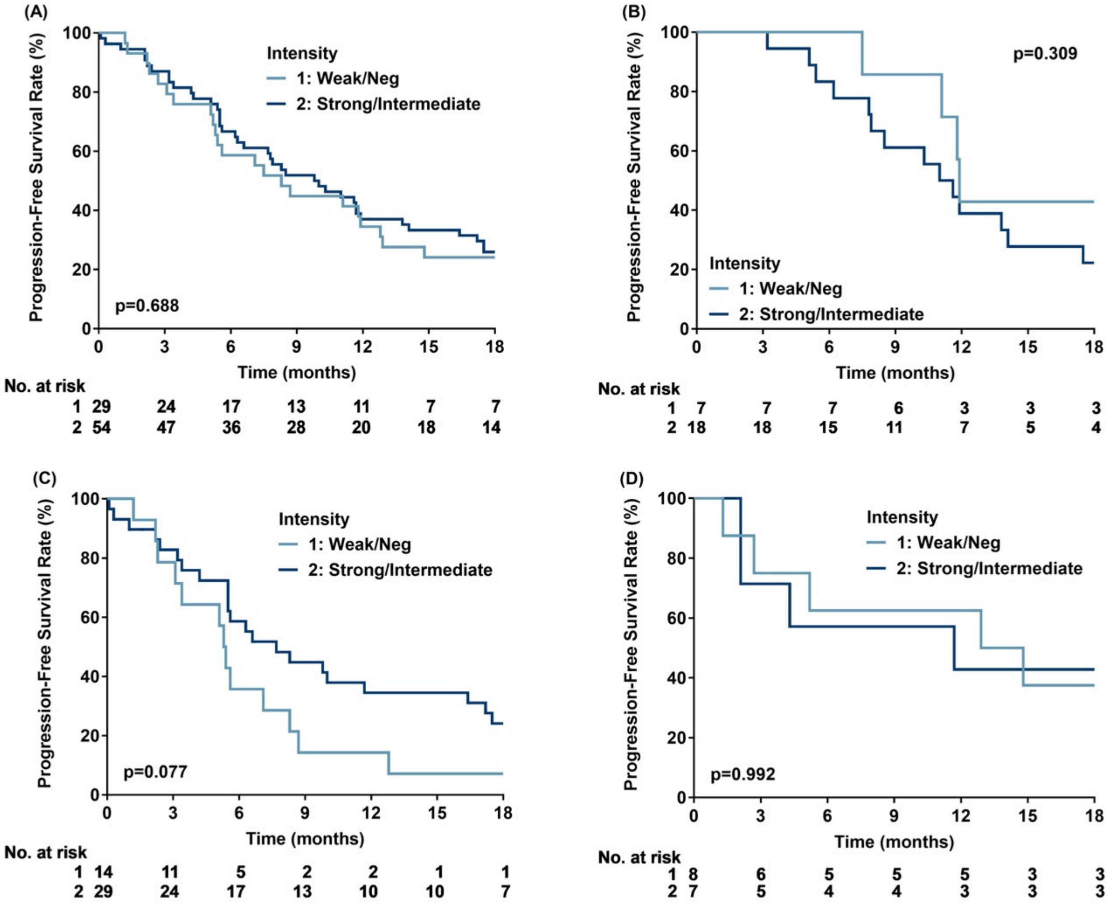

Progression-free survival based on p53 immunohistochemical analysis (strong/intermediate vs. weak/negative). A) All patients, B) patients harboring Del19, C) patients harboring L858R, and D) patients harboring other mutations.

Figure S3.

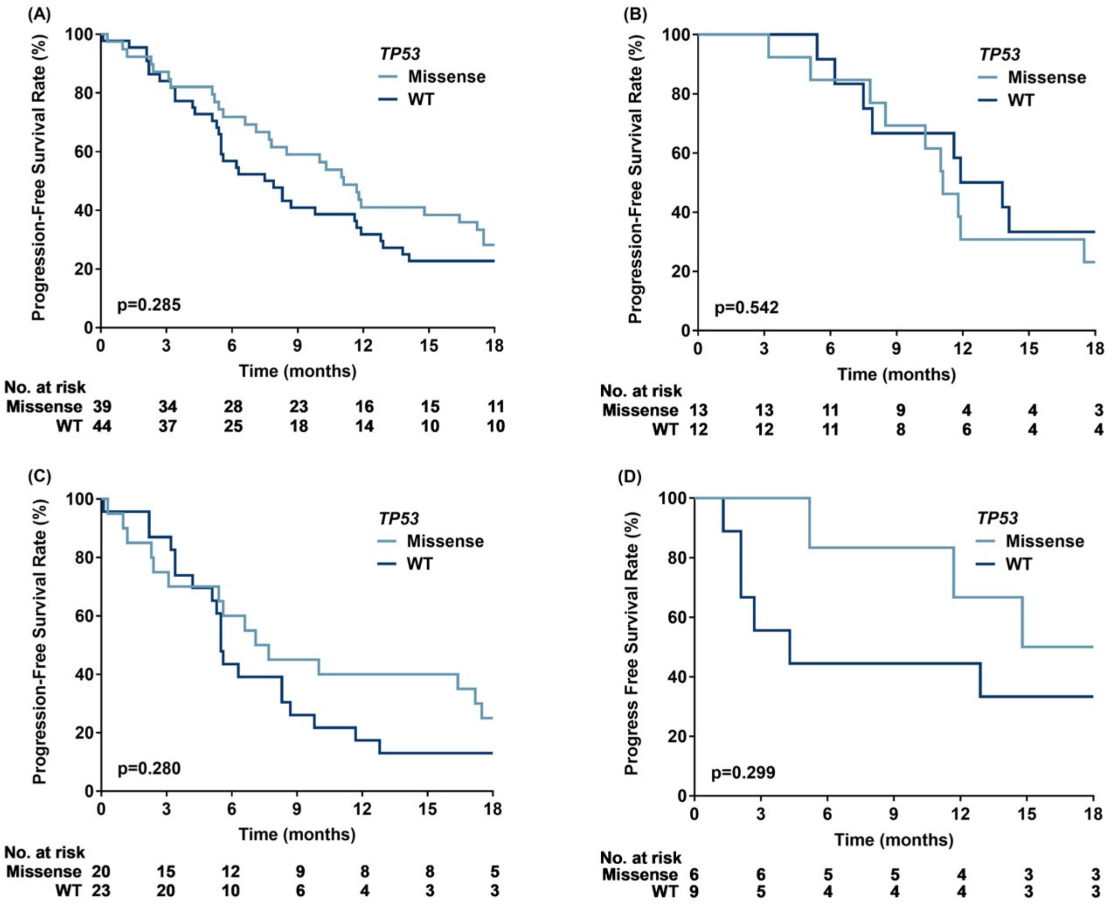

Progression-free survival based on *TP53* mutations (missense vs. WT). A) All patients, B) patients harboring Del19, C) patients harboring L858R, and D) patients harboring other mutations.

Figure S4.

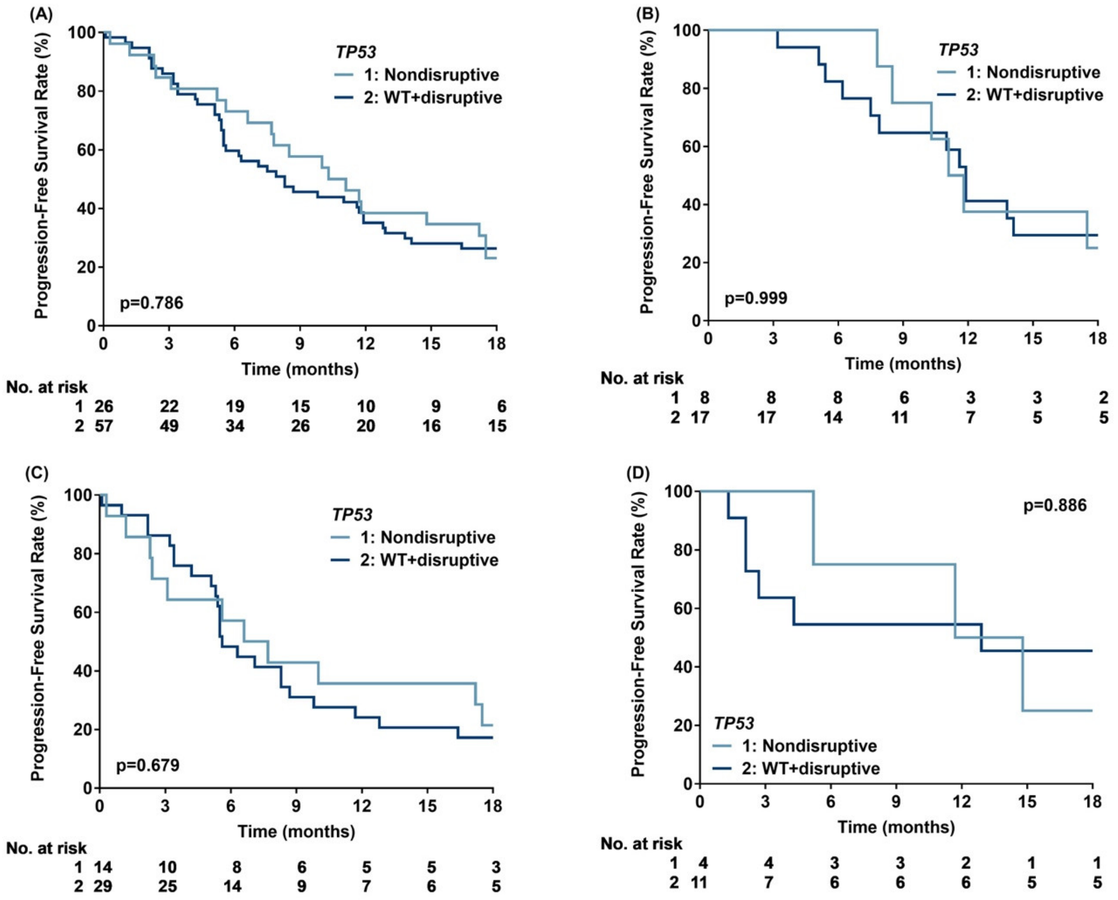

Progression-free survival based on *TP53* mutation (nondisruptive vs. WT+ disruptive). A) All patients, B) patients harboring Del19, C) patients harboring L858R, and D) patients harboring other mutations.

Figure S5.

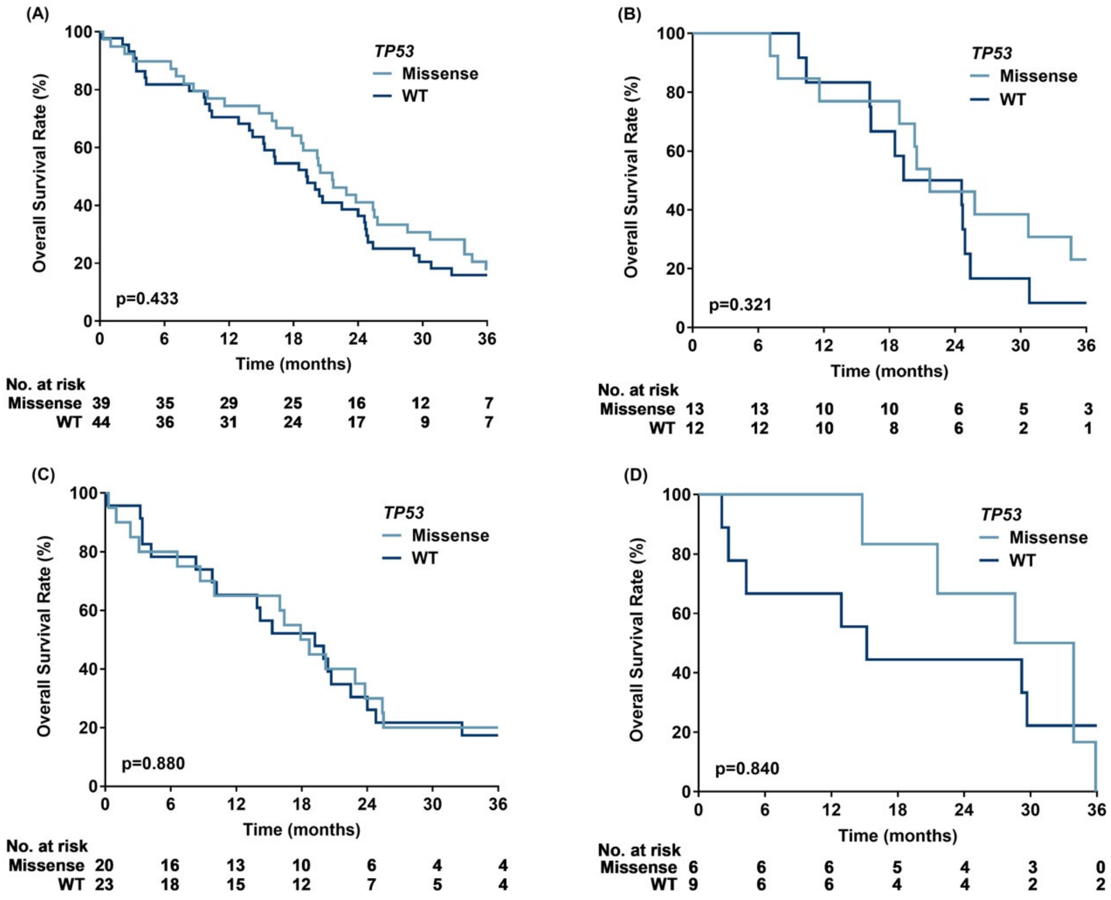

Overall survival based on *TP53* mutation (missense vs. WT). A) All patients, B) patients harboring Del19, C) patients harboring L858R, and D) patients harboring other mutations.

Figure S6.

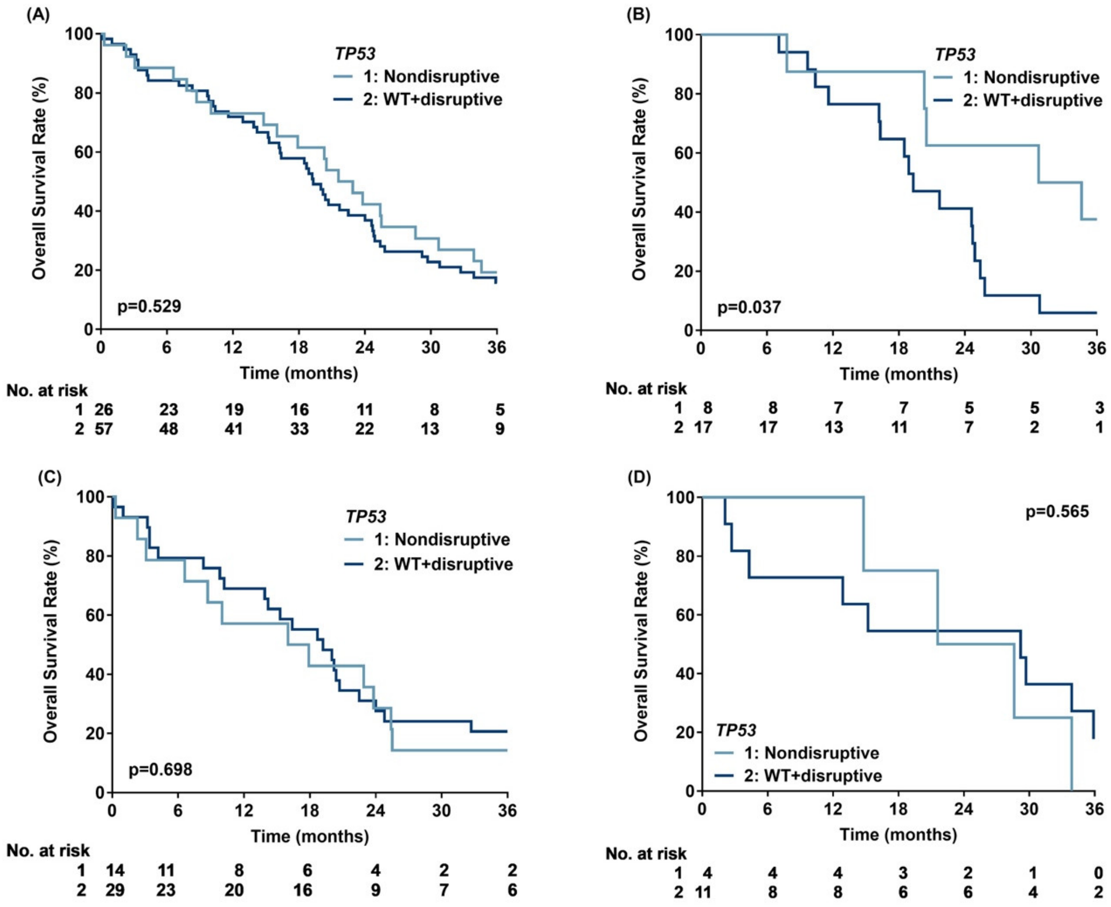

Overall survival by *TP53* mutation (nondisruptive vs. WT+ disruptive). A) All patients, B) patients harboring Del19, C) patients harboring L858R, and D) patients harboring other mutations.

Table S1. Semi-quantitative definition of p53 immunohistochemical staining based on staining intensity.

Score	0	1	2	3	4
Staining intensity	No staining	Weak	Intermediate		Strong
Proportion of positive tumor cells	0%	<10%	11%–49%	50%–80%	>80%

Table S2. Primers used for *TP53* genotyping.

Exon	Primers 5′- 3′	
TP53 EXON 5A	F	CACTTGTGCCCTGACTTTCA
	R	AGCCATGGCACGGACGCG
TP53 EXON 5B	F	CTCCTGCCCGGCACCCGC
	R	CTAAGAGCAATCAGTGAGGAATCAGA
TP53 EXON 6	F	AGACGACAGGGCTGGTTGC
	R	CAACCACCCTTAACCCCTCCT
TP53 EXON 7	F	CTTGGGCCTGTGTTATCTCC
	R	GGGTCAGAGGCAAGCAGA
TP53 EXON 8	F	GACCTGATTTCCTTACTGCCTCTTG
	R	AATCTGAGGCATAACTGCACCCTT

Table S3. Amplification conditions for *TP53* genotyping.

	HRM		Standard PCR	
EXON	Annealing time	Annealing temperature and cycles	Annealing time	Annealing temperature
5A	5 s	65°C–55°C touchdown 1°C/cycle for 10 cycles	30 s	55°C
5B	5 s	65°C–60°C touchdown 0.5°C/cycle for 10 cycles	30 s	60°C
6	5 s	68°C–58°C touchdown 1°C/cycle for 10 cycles	30 s	59°C
7	5 s	65°C–60°C touchdown 0.5°C/cycle for 10 cycles	30 s	60°C
8	20 s	63.5°C–58.5°C touchdown 0.5°C/cycle for 10 cycles	30 s	62°C

Table S4. Cross-tabulation of p53 immunohistochemical positivity (%) and missense *TP53* mutations.

	WT		Missense		
Variable	N	(%)	N	(%)	p-value
p53 positivity (%)					0.838
<10	11	(25.0)	9	(23.1)	
≥10	33	(75.0)	30	(76.9)	
p53 positivity (%)					0.260
<40	20	(45.5)	13	(33.3)	
≥40	24	(54.5)	26	(66.7)	
p53 positivity (%)					0.413
<50	22	(50.0)	16	(41.0)	
≥50	22	(50.0)	23	(59.0)	
